# A Few Things All Doctors Should Know about Bacterin Therapy

**Published:** 1917-01

**Authors:** C. W. Canan

**Affiliations:** Orkney Springs, Va.


### A FEW THINGS ALL DOCTORS SHOULD KNOW ABOUT BACTERIN THERAPY.

By C. W. Canan, M. D., B. S., Orkney Springs, Va.

With the rapid advance of vaccine and serum therapy into everyday practice the doctor's education is not complete without at least a general knowledge of its technique. To those who have not kept up with this branch of therapy, there is in store for them a great surprise when they must realize that at least one-half of all the diseases to which flesh is heir, can be treated by vaccine and serum therapy. And the results obtained are far more promising than by the old method of drugs. To be able to administer bacterins successfully certain accessories are necessary and a rigid antiseptic precaution must be kept in mind at every step of the procedure.

Bacterins and serums are all administered hypodermically, by injecting the liquid into the subcutaneous areolar tissue. What we have to say in reference to the kind of syringe to be used in this work and caring for the same, the reader can apply to the hypodermic administration of any and all class of remedies. The syringe best suited for this work and one that can be kept aseptic with the least labor, is an all-glass syringe, with a perfectly fitting glass plunger ground into the barrel—so that no packing of any kind, not even of asbestos, is necessary.

Since the writer has become familiar with the all-glass syringe he could never feel perfectly safe in using one of the older makes in which leather, rubber or even asbestos are used for packing, for fear of producing a "hypodermic abscess."

With the all-glass syringe the doctor will find that absolute asepsis is easily secured, and he need have no fear of infecting his patient if a few simple precautional measures are carried out.

In selecting a needle, especially for the introduction of bacterins, always choose platinum, because the salt solution in which the killed bacterins are suspended, in a bacterin will soon corrode and rust those made of steel while the platinum is not affected by it. For the administration of bacterin or serums the syringe to be convenient should hold 2 c. c., the needle should be one and one-half inches long, with size accordingly. In giving ordinary drugs a syringe of less capacity and a shorter needle will be found best.

The needle should be of the ''slip'' variety, into which the tip of the barrel has been ground so that a perfect union is secured, without the use of washers, gaskets, etc. The reader will readily understand that all these precautionary measures make for strict asepsis. A very extraordinary precaution that should never be omitted in administering either drugs or bacterins and with whatever make of syringe; is to always, before and after making an injection to fill the barrel of the syringe with alcohol and eject it through the needle; now wipe the needle thoroughly clean with gauze or cotton wet with alcohol not less than 92 per cent. In administering bacterins the site of the injection now claims our attention. In adults the most favorable site is, at a point over the insertion of the deltoid muscle, on the outer aspect of the upper arm. In children, especially very small ones, the buttock is the most convenient site. One reason for this choosing, is that when the child is turned face down across the nurse's lap, cannot see what is being transacted, also the tissues are looser here than in the arm, this makes the reaction less severe at the point of injection. In patients suffering from local infection the injection should be made if possible distal to the site of infection so that the newly formed antibodies may travel ''up-stream'' as Wright says to the ''seat of war.''

Should you find this impracticable make the injection in healthy tissue a little provimal to the focus of infection; in this way toxins will be nutralized and the bacteria destroyed before they can contaminate the blood stream. In treating diseases like peritonitis, appendicitis, puerperal infection, etc., the best site is about the middle of the inner aspect of the thigh. Never make the injection over bony prominences, or in close proximity with large nerves.

After the site for the injection has been selected, the skin should be thoroughly cleansed with alcohol. Should dirt be visible the skin should be scrubbed with warm water and soap before the use of the alcohol. Under certain conditions it is good policy to paint the site with Tr. Iodine immediately before the injection. The needle is now dextriously inserted for about one inch, it is then withdrawn about half this distance before the injection is made, so as to obviate the danger of introducing the bacterins into a blood vessel. Remember in this connection, that the injection is to be made into the subcutaneous tissue and not into the intramuscular tissue. It is claimed that the connective tissue cells have a much greater antibody producing power than the muscular cells.

The ablest authorities on this subject claim that they get better results, if a large dose is to be given, to divide it and make the injection into two or more sites.

One thing is certain, by dividing the dose, and injecting at several points, the local reaction will be less severe. The doctor should inform the patient after the first injection, that in all probability within twenty-four hours a red-zone and swelling will occur at the site of injection, but they should not be alarmed, that it will all subside in two or three days.

In a few instances a small hard palpabe nodule remains for quite a time, after the acute reaction has subsided, but this, too, will eventually disappear.

In reference to the size dose, the writer would advise that the first dose of bacterin should not be too large, rather depend upon the minimum dose of that special bacteria to be administered because of a possible idiosincrasy of the patient. If there is no response in 24 to 48 hours, the dose then can be increased with safety. There is always more or less reaction after a full medicinal dose of bacterin. The doctor should be posted upon this point, that he may be able to explain to his patient what these constitutional symptoms signify· They are evidence of either a **negative** or **positive** phase.

The clinical evidence of the **negative phase** are as follows: Within a few hours after the injected bacterin the temperature rises from a fraction of a degree to one or more degrees, the pulse becomes somewhat excelerated, chilly sensations may be complained of; also headache and a general malaise. These symptoms come on as a rule a few hours after the dose of bacterin has been administered and are known as the **negative phase.**

Ordinarily this phase lasts only a few hours, and is followed by a feeling of buoyancy or well-being and there is observed a pronounced amelioration of all the clinical symptoms of the disease from which the patient is suffering. This is the **positive phase.**

Upon the **negative** and **positive phase** we must base our judgment as to the size of dose, and the time of its administration, to a certain degree at least. The size of the dose of a bacterin may be too large or too small or may be administered too close together or too far apart, consequently, be disappointing.

It is stated by those best qualified to give advice that too small doses are employed more often than too large ones.

Again, the intervals between doses may not be properly spaced. We must not administer the doses so close together, that we have what is termed a **cumulative negative phase.** Always wait until the negative phase has passed and the **positive phase** is manifesting itself before repeating the next dose. In other words, wait until there is an improvement in the clinical symptoms, and a feeling of well-being to the patient, him or herself.

We have not mentioned the opsonic index as a guide to the size of dose and the time at which it should be repeated, because of the intricate procedure necessary to obtain the opsonic index. In urgent, acute conditions like those of pneumonia, septicaemia it is best to repeat small doses every twelve to twenty-four hours until improvement is manifested.

In tubercular infection small doses of tubercle-bacterin should be employed every ten to twelve days.

A word, in conclusion, in reference to stock bacterin in comparison with autogenous bacterin as to their curative value. This question is still undecided. In some cases stock bacterins will fail to produce the desired results, while the employment of autogenous products will often result in a brilliant cure. In other instances the reverse is true, we get good results with stock bacterin in cases where the autogenous variety have failed.

The writers experience has been in favor of the autogenous type in all chronic skin troubles and in fact chronic conditions in general.

